# Association Between Specialist Office Visits and Health Expenditures in Accountable Care Organizations

**DOI:** 10.1001/jamanetworkopen.2019.6796

**Published:** 2019-07-10

**Authors:** Vishal Anand Shetty, Laura B. Balzer, Kimberley H. Geissler, David L. Chin

**Affiliations:** 1 Department of Health Policy and Promotion, University of Massachusetts Amherst, Amherst

## Abstract

**Importance:**

Accountable care organizations (ACOs) aim to control health expenditures while improving quality of care. Primary care has been emphasized as a means to reduce spending, but little is known about the implications of using specialists for achieving this ACO objective.

**Objective:**

To examine the association between ACO-beneficiary office visits conducted by specialists and the cost and utilization outcomes of those visits.

**Design, Setting, and Participants:**

This cross-sectional study obtained data on 620 distinct ACOs from the Centers for Medicare & Medicaid Services Shared Savings Program Accountable Care Organizations Public-Use Files from April 1, 2012, to September 30, 2017. Generalized estimating equation models were used for analysis of ACOs, adjusting for ACO-beneficiary health status, Medicare enrollment groups, ACO size, and proportion of participating specialists.

**Exposures:**

Specialist encounter proportion, the percentage of office visits provided by a specialist, was categorized into 7 discrete groups: less than 35%, 35% to less than 40%, 40% to less than 45% (reference group), 45% to less than 50%, 50% to less than 55%, 55% to less than 60%, and 60% or greater.

**Main Outcomes and Measures:**

The primary outcome was total expenditures (given in US dollars) per assigned beneficiary person-year. The secondary outcomes were total numbers of emergency department visits, hospital discharges, skilled nursing facility discharges, and magnetic resonance imaging orders.

**Results:**

In total, the data set included 1836 ACO-year (number of participation years per ACO) observations for 620 distinct ACOs. Those ACOs with a specialist encounter proportion of 40% to less than 45% had $1129 (95% CI, $445-$1814) lower per-beneficiary person-year spending than did ACOs in the lowest specialist encounter proportion group and had $752 (95% CI, $115-$1389) lower per-beneficiary person-year spending compared with ACOs in the highest specialist encounter proportion group. Monotonic decreases in emergency department visits, hospital discharges, and skilled nursing facility discharges were observed with increasing specialist encounter proportion. Conversely, monotonic increases in magnetic resonance imaging volume discharges were observed with increasing specialist encounter proportion.

**Conclusions and Relevance:**

These findings suggest that an ACO’s ability to reduce spending may require sufficient involvement in care processes from specialists, who seem to complement the intrinsic primary care approach in ACOs.

## Key Points

**Question:**

What is the association between office visits conducted by specialists and health care spending in an accountable care organization?

**Findings:**

In this cross-sectional study of 620 distinct accountable care organizations, organizations in which 40% to 45% of patient visits were provided by specialists had statistically significantly lower per-beneficiary person-year spending compared with those in which less than 35% or at least 60% of the visits were conducted by specialists.

**Meaning:**

Some specialist involvement in care processes for patients appears to be necessary for accountable care organizations to lower their costs.

## Teaser

This cross-sectional study examines US Medicare data to assess variations in health care costs and utilization between accountable care organization practices that rely heavily on specialty physicians and those that only use primary care physicians.

## Introduction

The Medicare Shared Savings Program (MSSP) accountable care organization (ACO) is a health care payment and delivery model intended to incentivize a consortium of health care practitioners who control spending by cooperating, communicating, and coordinating patient care across multiple clinical settings.^1^ By creating shared accountability and incentives for clinicians, ACOs may improve patient outcomes while lowering costs. The Centers for Medicare & Medicaid Services establishes a financial benchmark before each agreement period with ACOs that is based on fee-for-service payments for ACO beneficiaries during the previous 3-year period; this benchmark is prospectively reestimated each year of participation. If an MSSP ACO’s expenditures are less than this risk-adjusted benchmark and it fulfills specific quality measure objectives, the ACO receives a financial payment equal to a proportion of the savings.

In April 2017, more than 500 Medicare ACOs provided care to nearly 10 million people.^2^ The putative success of the MSSP ACOs depended largely on successfully incentivizing strong organizational leadership and expanding the responsibilities of primary care physicians (PCPs) to coordinate care,^3,4^ approaches believed to be necessary to slow the growth of spending.^5^ With the broad implementation of the ACO payment model, studies have reported modest improvements in reducing expenditures and enhancing quality of care.^6,7^ Given that most health expenditures are associated with care for a small proportion of patients with complex clinical conditions,^8^ specialists may play an important role in containing costs for patients with high-resource needs.^9^ Specialists can also support judicious service use, when aligned with ACO incentives, across the ACO patient population.^10^ The MSSP does not require specialist membership for ACO formation,^11^ but previous studies suggest that the integration of specialists in an ACO may be financially advantageous.^12,13^

Because of the short period since ACOs were first implemented,^1^ evidence is currently limited on the use of care from specialists by ACO enrollees, although some patterns have emerged. Those ACOs composed predominantly of PCPs may be unable to satisfy their patients’ specialty care needs without depending on external specialists.^14^ This situation may limit an ACO’s ability to control costs, which may be associated with these PCP-oriented ACOs having reductions in the overall use of specialty care.^15^ Conversely and as expected, ACOs composed predominantly of specialists, when compared with ACOs with a nonspecialist majority, appear to have successfully contained specialty care within the ACO ^14^ but have not decreased the overall use of specialty care.^15^

An early study of ACOs examined the association between office visits from PCPs and health care spending and utilization rates.^10^ These investigators found that ACOs that provided the least number of office visits through PCPs had lower hospital admissions and emergency department (ED) visits but similar spending levels when compared with ACOs that provided the highest number of office visits through PCPs. Although the present study shares some insight into the association of specialists with ACOs, it did not examine ACO spending when PCP and specialist involvement levels were balanced.

Using 5 years of data on MSSP ACOs, we examined the association between the proportion of office visits conducted by specialists and spending in ACOs. We hypothesized that the highest expenditures would be among ACOs with the smallest and greatest percentages of specialist-conducted patient services. We also examined the association between the proportion of specialist-provided office visits and 4 measures of utilization to understand possible factors in spending.

## Methods

We used the Centers for Medicare & Medicaid Services Shared Savings Program Accountable Care Organizations Public-Use Files from April 1, 2012, to September 30, 2017, to conduct this analysis.^16^ These data represent ACO-level annual characteristics for the first 5 MSSP ACO performance periods from April 1, 2012, to December 31, 2017. The Human Research Protection Office at the University of Massachusetts determined the protocol to not be human subjects research, given that the data used in this study are publicly available, aggregated by ACO-year (number of participation years per ACO), and contain no patient-level information. This study followed the Strengthening the Reporting of Observational Studies in Epidemiology (STROBE) reporting guideline.

### Study Variables

The primary outcome was total expenditures per assigned beneficiary person-year in the performance year, as defined by the MSSP.^17^ We examined 4 secondary outcomes, all per 1000 person-years in the performance year: (1) total number of ED visits, (2) total number of inpatient hospital discharges, (3) total number of skilled nursing facility (SNF) discharges, and (4) total number of magnetic resonance imaging (MRI) orders. These secondary outcomes were established measures of utilization and used in previous ACO studies.^18,19^ The primary variable (eAppendix in the Supplement) was the specialist encounter proportion, defined as the percentage of office visits provided by a specialist. It was categorized into 7 mutually exclusive groups: less than 35.0%, 35% to less than 40%, 40% to less than 45% (the reference group), 45% to less than 50%, 50% to less than 55%, 55% to less than 60%, and 60% or greater. The upper and lower percentile bounds were selected to include a sufficient number of ACOs in each group. The specialist encounter proportion included office visits with all clinicians (both ACO affiliated and non–ACO affiliated).

We accounted for the following ACO characteristics: health status, Medicare enrollment groups, ACO size, and specialist participation. These characteristics were selected because they were factors associated with both the exposure (specialist encounter proportion) and the primary outcome (total expenditures).^10,15^ To calculate severity of illness in each ACO, we included variables that accounted for beneficiary eligibility categories and the relative health status of enrollees in each category. Specifically, we took the proportion of person-years for beneficiaries with end-stage renal disease, with disability, with dual-eligible status, or with non–dual-eligible status and multiplied those person-years by the mean hierarchical condition category (HCC) scores for each respective category.

The HCC scores reflected a risk-adjustment model, which captured patient health status as a measure of the estimated cost of caring for that patient in association with the mean cost within that clinical category.^20,21^ For each ACO, the Medicare enrollment groups (persons with end-stage renal disease, with disability, with dual-eligible status, and with non–dual-eligible status) had a corresponding HCC score. The HCC score, combined with demographic characteristics, represented the severity of illness within that subgroup. The ACO size was measured by the total beneficiary person-years in the performance year. Beneficiaries in the dual-eligible status group were those who qualified for both Medicare and Medicaid (only Medicare for beneficiaries with non–dual-eligible status). These groups were included as a measure of the socioeconomic status of each ACO’s beneficiary population. Specialist participation was measured by dividing the number of participating specialists in an ACO by the total number of participating clinicians.

### Statistical Analysis

We calculated summary statistics for the outcome and covariates within each specialist encounter proportion group. Generalized estimating equation (GEE) models^22^ with an exchangeable correlation matrix and robust variance estimators were used to estimate the association between ACO expenditures and specialist encounter proportion while accounting for health status, Medicare enrollment groups, ACO size, and specialist participation; these GEE models accounted for within-ACO correlation. For the secondary outcomes, we used GEE models to estimate the association between the 4 utilization measures and specialist encounter proportion, adjusting for the same covariates as in the primary analysis. A 2-sided *P* < .05 was considered significant. All statistical analyses were conducted using R, version 3.3.1, and the package geepack^23^ (R Project for Statistical Computing) was used for GEE models.

## Results

The analytic data set included 1836 ACO-year (number of participation years per ACO) observations for 620 distinct ACOs. Summary statistics are presented for the lowest (<35%), reference (40% to <45%), and highest (≥60%) primary variable groups and the entire cohort of ACO-year observations (Table; eTable 1 in the Supplement). Compared with ACOs with a specialist encounter proportion between 40% and 45%, ACOs with the lowest specialist encounter proportion had a mean 14.6% higher expenditures, and ACOs with the highest specialist encounter proportion had a mean 11.1% higher expenditures. In addition, ACOs with the lowest specialist encounter proportion differed from ACOs with the highest specialist encounter proportion across several characteristics. The ACOs with the lowest specialist encounter proportions had means of 41.0% fewer beneficiaries, 33.9% lower specialist participation, a 10.0% higher proportion of person-years for beneficiaries with a disability, and a 5.2% higher proportion of person-years for beneficiaries with a dual-eligible status compared with ACOs with high specialist encounter proportions.

**Table.** Descriptive Statistics of ACOs

Characteristic	Mean (SD)			
	Overall	Specialist Encounter Proportion Group, %^a^		
		<35	40 to <45	>60
No. of ACO-year observations^b^	1836	178	431	64
No. of unique ACOs	620	78	211	31
Total beneficiaries, No.	31 861 238	1 980 927	8 120 335	1 207 800
Expenditures per beneficiary, $	11 017 (2981)	11 975 (5611)	10 641 (2566)	12 465 (2351)
ACO size, No.	17 353 (17 046)	11 129 (8680)	18 841 (19 935)	18 871 (11 551)
Specialist participation, %^c^	40.5 (20.5)	29.2 (20.2)	39.7 (19.5)	63.1 (11.5)
Proportion of person-years for beneficiaries with ESRD, %	1.0 (0.6)	1.0 (0.9)	1.0 (0.6)	1.0 (0.6)
Proportion of person-years for beneficiaries with disability, %	14.2 (7.2)	20.3 (9.0)	14.7 (6.3)	10.3 (4.2)
Proportion of person-years for beneficiaries with dual-eligible status, %	8.4 (9.7)	13.1 (10.5)	9.0 (11.8)	6.9 (4.0)
Proportion of person-years for beneficiaries with non–dual-eligible status, %	76.4 (13.6)	65.6 (15.9)	75.3 (13.9)	81.8 (7.5)
HCC score^d^				
ESRD	1.02 (0.06)	1.03 (0.08)	1.02 (0.05)	1.03 (0.07)
Disability	1.09 (0.14)	1.05 (0.22)	1.09 (0.12)	1.15 (0.20)
Dual eligible	1.03 (0.12)	1.01 (0.14)	1.02 (0.11)	1.06 (0.08)
Non–dual eligible	1.06 (0.11)	1.07 (0.21)	1.05 (0.10)	1.08 (0.08)

Abbreviations: ACO, accountable care organization; ESRD, end-stage renal disease; HCC, hierarchical condition category.

^a^ Specialist encounter proportion is the proportion of office visits provided by a specialist.

^b^ ACO-year is the number of participation years per ACO.

^c^ Specialist participation is the proportion of clinicians participating in the ACO who were specialists.

^d^ Higher HCC scores indicate lower health status; the HCC risk scores for each enrollment type are renormalized to their own populations and thus are not on the same scale and not comparable across eligibility types.

**Figure 1.**
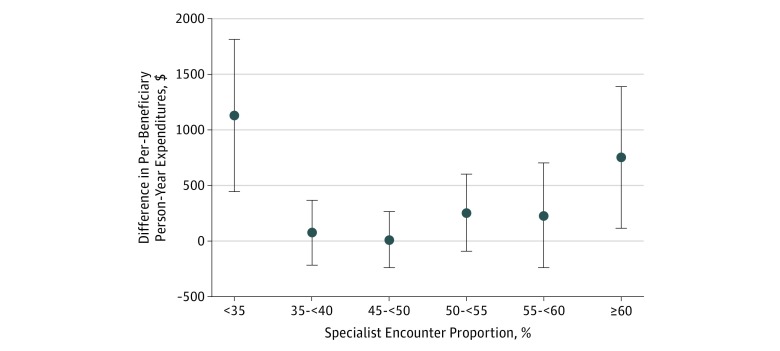
Adjusted Association Between Expenditures and Specialist Encounter Proportion Groups^a^ Data markers represent differences in per-beneficiary person-year spending between each specialist encounter proportion group and the reference group (40% to <45%). Error bars indicate 95% CIs, with those crossing $0 not statistically significant. All results were regression adjusted for health status, Medicare enrollment groups, accountable care organization size, and specialist participation. ^a^Specialist encounter proportion is the proportion of office visits provided by a specialist.

The ACOs with the lowest and highest specialist encounter proportions had the highest expenditures. When compared with the reference group (40% to <45%), ACOs in the lowest specialist encounter proportion group spent $1129 (95% CI, $445-$1814) and those in the highest spent $752 (95% CI, $115-$1389) more per capita (Figure 1). Expenditures were incrementally higher for the group with the higher specialist encounter proportion than in the reference group, although these results were not statistically different across groups. These trends were similar in the unadjusted results (eTable 2 in the Supplement).

**Figure 2.**
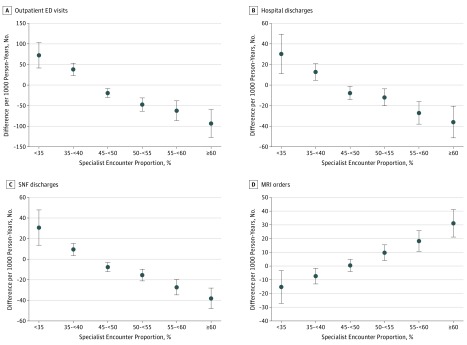
Adjusted Association Between Utilization Outcomes and Specialist Encounter Proportion Groups Data markers represent differences in utilization measures between each specialist encounter proportion group and the reference group (40% to <45%). Error bars indicate 95% CIs, with those crossing 0 not statistically significant. All results were regression adjusted for health status, Medicare enrollment groups, accountable care organization size, and specialist participation. Specialist encounter proportion is the proportion of office visits provided by a specialist. ED indicates emergency department; MRI, magnetic resonance imaging; and SNF, skilled nursing facility.

The adjusted use models showed ED visit, hospital discharge, and SNF discharge rates monotonically decreased as specialist encounter proportion increased; MRI order rates monotonically increased as the encounter proportion decreased (Figure 2). Compared with ACOs in the reference group, ACOs in the lowest specialist encounter proportion group had 72.1 (95% CI, 41.4-102.8) more ED visits, 30.2 (95% CI, 11.0-49.4) more hospital discharges, and 30.7 (95% CI, 13.6-47.8) more SNF discharges per 1000 person-years. Meanwhile, ACOs in the highest specialist encounter proportion group had 93.2 (95% CI, –127 to –59.2) fewer ED visits, 35.9 (95% CI, –51.3 to –20.4) fewer hospital discharges, and 38.1 (95% CI, –48.1 to –28.1) fewer SNF discharges per 1000 person-years. The trend for MRI orders was reversed with ACOs in the lowest specialist encounter proportion group, with 15.3 (95% CI, –27.2 to –3.3) fewer MRI orders per 1000 person-years compared with ACOs in the reference group. Compared with referent ACOs, ACOs in the highest specialist proportion group had 31.1 (95% CI, 9.7-52.5) more MRI orders per 1000 person-years.

## Discussion

During the first 5 performance years of the MSSP, we found that expenditures were lowest for ACOs with a balanced specialist encounter proportion (40% to <45%), whereas ACOs at the specialist encounter proportion extremes (<35% and ≥60%) had the highest expenditures. Those ACOs with the lowest specialist encounter proportion had the highest rates of ED visits, hospital discharges, and SNF discharges as well as the lowest MRI volume; in contrast, ACOs with the highest specialist encounter proportion had the highest MRI volume but the lowest rates of ED visits, hospital discharges, and SNF discharges. These findings suggest that ACOs that provide office visits through a balance of PCPs and specialists may be better positioned to achieve utilization rates that are consistent with lower costs, compared with ACOs that provide office visits through a more skewed PCP and specialist distribution.

Most studies of ACO performance (in both the MSSP and Pioneer programs) have compared the differences in spending between ACO-affiliated and non–ACO-affiliated clinicians.^6,7,18,19^ These studies have consistently found that variations in aggregate spending could be associated with decreases in spending on inpatient, outpatient, and postacute care. One study found that ACOs in the lowest decile of spending per beneficiary in 2015 had lower inpatient admissions than ACOs in the highest decile of spending.^24^ It also found that ACOs with the lowest spending had lower SNF admissions compared with ACOs with the highest spending. The associations of lower spending with lower inpatient volume and lower SNF utilization were consistent with our findings when comparing ACOs in the referent specialist encounter proportion group with ACOs in the lowest specialist encounter proportion group. However, these associations were inconsistent with our findings when comparing ACOs in the referent specialist encounter proportion group with ACOs in the highest specialist encounter proportion group.

In contrast to the findings in the present study, Herrel et al^10^ found fewer ED, hospital, and SNF discharges in ACOs in the lowest quartile of primary care focus (ACOs that provided the fewest office visits by PCPs) when compared with ACOs in the highest quartile of primary care focus (ACOs that provided the most office visits by PCPs), as well as no spending differences between these ACO groups. The difference in findings between the Herrel et al^10^ study and the present study may be associated with variable definitions (office visits from specialists vs from PCPs), selection of comparison groups (7 discrete groups across the range of the variable vs the lowest and highest quartiles of the variable), and an increased number of years of ACO performance data.

Differences in spending between ACOs may be associated with variations in incentives at the physician or facility level. As illustrated in a previous study, physician group–owned ACOs were able to achieve significantly greater reductions in spending than hospital-integrated ACOs from 2012 to 2015, when both groups were compared with local non–ACO-affiliated groups.^18^ The ACOs integrated with acute care facilities may have less financial incentive to reduce inpatient spending when compared with ACOs established on an outpatient, physician group–based practice if bonuses from shared savings do not compensate for lost fee-for-service revenue.^25^ In addition, the ACO’s market share may be a factor in its specialist composition, incentives, and behavior.^26,27,28^ Similar to large hospitals, ACOs with a large market share may have a greater ability to choose their specialist composition and provide a higher level of control over the affiliated specialists.^28^

The finding of higher expenditures among ACOs with a high specialist encounter proportion suggests that financial incentives for specialists to maximize clinical volume may be more a factor than ACO-level incentives in reducing spending, which aligns with the conclusions of several previous studies.^6,12,18^ However, based on our findings, the results among ACOs with the lowest specialist encounter proportion appear to not follow this trend. The finding of high expenditures among ACOs with the lowest specialist encounter proportion suggests patients in this group received outpatient care (predominantly delivered by PCPs) associated with higher ED, hospital, and SNF encounter rates. Although PCPs may play an important role in the coordination and management of patient care, particularly for those with chronic illness,^29^ active specialist involvement in the continuum of patient care (eg, acute diagnosis consultations, specialist-provided procedures, or comanagement of complex conditions) may be important in improving outcomes.^30,31^ Our findings also suggest some specialist involvement in outpatient care may be associated with lower spending for ACOs, but whether this decrease leads to a trade-off in quality is unclear. Future research is needed to understand the implications of ACO characteristics associated with spending reductions for quality of care.

Despite having specialist membership, some ACOs were heavily reliant on PCPs to deliver ambulatory care. However, factors outside the control of the ACOs, such as PCPs providing more comprehensive care or PCPs being given referral incentives established before ACO formation, may be associated with the imbalanced volume of specialist membership to office visits. Clinician composition is considered to be an important factor in establishing utilization patterns in ACOs.^15^ However, we found that composition was only moderately associated with specialist encounter proportion. Our adjustment of composition was intended to account for any differences in incentives based on the makeup of clinicians affiliated with the ACO, although the association between spending and specialist encounter proportion did not change after the adjustment. This outcome indicates that patterns of care may be more closely associated with ACO expenditures than the composition of participating clinicians, particularly given the high rates of leakage (proportion of care delivered outside of the ACO) of specialty care to external clinicians.^15^

Nevertheless, ACOs are responsible for the costs incurred for their patients across the spectrum of care, yet some specialists participating in ACOs have little involvement in ACO decision-making.^12^ Policy makers may consider amendments to increase the accountability of specialists for ACO objectives, such as mandatory specialist representation in ACO governance and beneficiary attribution through specialists. Future empirical studies are needed into which structural components and processes of care coordination between PCPs and specialists are associated with the lowest ACO costs and improvement in the quality of care.

### Limitations

This study has several limitations. First, the generalizability of these findings may be limited to MSSP ACOs. Accountable care organizations have been implemented by a variety of payers in different contexts, such as state Medicaid ACO programs, Medicare Pioneer, and private insurers. These various ACO models may have similarities, but the MSSP has its own set of structural conditions and requirements that may not apply to other types of ACOs. However, MSSP ACOs represent more than 50% of all ACOs,^32^ and they account for less than 30% of lives covered by any ACO.^2^ Second, we were unable to distinguish office visit encounters with ACO-affiliated clinicians from non–ACO-affiliated clinicians, and we could not quantify the amount of leakage that occurred. However, we adjusted for clinician composition, a primary factor in potential differences in leakage between ACOs,^15^ and no evidence suggested leakage varied across specialist encounter proportion groups.

Third, we were unable to account for ownership status, market share, or rurality of the ACOs, factors that may be associated with unmeasured confounders.^6,25,33^ However, we controlled for ACO characteristics associated with size, Medicare eligibility, and health status, which were expected factors in both the specialist encounter proportion and expenditures. We also noted the potential for greater HCC variation among newly formed ACOs, but the inclusion of ACO experience (years since formation) as an adjustment variable did not meaningfully change this study’s results. Fourth, these analyses were limited to ACO-level implications. Future studies of within-ACO implications, such as the type of specialist care delivered and patient-specific costs, may require patient encounter–level information.

## Conclusions

Health policy makers have consistently emphasized the important role of PCPs in coordinating care for patients and in resource stewardship to achieve spending reductions, but less attention has been given to specialists’ role in achieving these goals. We found that ACOs that provided 40% to less than 45% of office visits through specialists had statistically significantly lower expenditures compared with ACOs with the lowest and highest specialist encounter proportion, after controlling for health status, Medicare enrollment groups, ACO size, and specialist participation. In addition, ACOs with the lowest specialist encounter proportion had higher relative ED visits, hospital discharges, and SNF discharges than ACOs that provided office visits through a comparable proportion of specialists and nonspecialists.

Establishing a foundation of primary care may be a necessary first step for ACOs to meet their objectives, but sustained reductions in spending for Medicare may not be achievable without sufficient involvement in the care processes by specialists. Future studies of the ACO payment model are needed to understand specialists’ role in the continuum-of-care coordination associated with improved health outcomes and lower cost of care.

## Notes

Supplement. **eAppendix.** Explanation of Primary Predictor **eTable 1. **Accountable Care Organization Characteristic Descriptive Statistics, All Groups **eTable 2. **Per-Beneficiary Person-Year Expenditure Differences Between Specialist Encounter Proportion Groups and Reference (40% to <45%)

## References

[1] Centers for Medicare & Medicaid Services. Accountable Care Organizations (ACOs) https://www.cms.gov/Medicare/Medicare-Fee-for-Service-Payment/ACO/. Updated March 8, 2019. Accessed June 1, 2019.

[2] Muhlestein D, Saunders R, McClellan M Growth of ACOs and alternative payment models in 2017. Health Affairs Blog. https://www.healthaffairs.org/do/10.1377/hblog20170628.060719/full/. Published June 28, 2017. Accessed June 1, 2019.

[3] Rittenhouse DR, Shortell SM, Fisher ES Primary care and accountable care–two essential elements of delivery-system reform. N Engl J Med. 2009;361(24):-. doi:10.1056/NEJMp0909327 19864649

[4] McClellan M, McKethan AN, Lewis JL, Roski J, Fisher ES A national strategy to put accountable care into practice. Health Aff (Millwood). 2010;29(5):982-990. doi:10.1377/hlthaff.2010.0194 20439895

[5] McWilliams JM, Chernew ME, Zaslavsky AM, Hamed P, Landon BE Delivery system integration and health care spending and quality for Medicare beneficiaries. JAMA Intern Med. 2013;173(15):1447-1456. doi:10.1001/jamainternmed.2013.6886 23780467 PMC3800215

[6] McWilliams JM, Hatfield LA, Chernew ME, Landon BE, Schwartz AL Early performance of accountable care organizations in Medicare. N Engl J Med. 2016;374(24):2357-2366. doi:10.1056/NEJMsa1600142 27075832 PMC4963149

[7] McWilliams JM, Chernew ME, Landon BE, Schwartz AL Performance differences in year 1 of pioneer accountable care organizations. N Engl J Med. 2015;372(20):1927-1936. doi:10.1056/NEJMsa1414929 25875195 PMC4475634

[8] Cohen SB. Differentials in the concentration of health expenditures across population subgroups in the U.S., 2012. https://meps.ahrq.gov/data_files/publications/st448/stat448.shtml. Published September 2014. Accessed June 1, 2019.

[9] Powers BW, Chaguturu SK ACOs and high-cost patients. N Engl J Med. 2016;374(3):203-205. doi:10.1056/NEJMp1511131 26789867

[10] Herrel LA, Ayanian JZ, Hawken SR, Miller DC Primary care focus and utilization in the Medicare shared savings program accountable care organizations. BMC Health Serv Res. 2017;17(1):139. doi:10.1186/s12913-017-2092-8 28202052 PMC5311837

[11] Centers for Medicare & Medicaid Services. ACO Participant List and Participant Agreement Medicare Shared Savings Program. https://www.cms.gov/Medicare/Medicare-Fee-for-Service-Payment/sharedsavingsprogram/Downloads/ACO-Participant-List-Agreement.pdf. Published April 2019. Accessed June 1, 2019.

[12] Dupree JM, Patel K, Singer SJ, Attention to surgeons and surgical care is largely missing from early medicare accountable care organizations. Health Aff (Millwood). 2014;33(6):972-979. doi:10.1377/hlthaff.2013.1300 24889946

[13] Hawken SR, Ryan AM, Miller DC Surgery and Medicare Shared Savings Program accountable care organizations. JAMA Surg. 2016;151(1):5-6. doi:10.1001/jamasurg.2015.2772 26509237 PMC4747022

[14] McWilliams JM, Chernew ME, Dalton JB, Landon BE Outpatient care patterns and organizational accountability in Medicare. JAMA Intern Med. 2014;174(6):938-945. doi:10.1001/jamainternmed.2014.1073 24756690

[15] Barnett ML, McWilliams JM Changes in specialty care use and leakage in Medicare accountable care organizations. Am J Manag Care. 2018;24(5):e141-e149. 29851445 PMC5986093

[16] Centers for Medicare & Medicaid Services Shared Savings Program Accountable Care Organizations (ACO) Public-Use Files. https://www.cms.gov/research-statistics-data-and-systems/downloadable-public-use-files/sspaco/index.html. Published October 27, 2017. Accessed March 17, 2018.

[17] Centers for Medicare & Medicaid Services Financial and beneficiary assignment specifications. https://www.cms.gov/Medicare/Medicare-Fee-for-Service-Payment/sharedsavingsprogram/Financial-and-Assignment-Specifications.html. Updated May 21, 2019. Accessed June 1, 2019.

[18] McWilliams JM, Hatfield LA, Landon BE, Hamed P, Chernew ME Medicare spending after 3 years of the Medicare Shared Savings Program. N Engl J Med. 2018;379(12):1139-1149. doi:10.1056/NEJMsa1803388 30183495 PMC6269647

[19] Nyweide DJ, Lee W, Cuerdon TT, Association of pioneer accountable care organizations vs traditional Medicare fee for service with spending, utilization, and patient experience. JAMA. 2015;313(21):2152-2161. doi:10.1001/jama.2015.4930 25938875

[20] Li P, Kim MM, Doshi JA Comparison of the performance of the CMS Hierarchical Condition Category (CMS-HCC) risk adjuster with the Charlson and Elixhauser comorbidity measures in predicting mortality. BMC Health Serv Res. 2010;10(1):245. doi:10.1186/1472-6963-10-245 20727154 PMC2936901

[21] Sukul D, Hoffman GJ, Nuliyalu U, Association between Medicare policy reforms and changes in hospitalized Medicare beneficiaries’ severity of illness. JAMA Netw Open. 2019;2(5):e193290-e193290. doi:10.1001/jamanetworkopen.2019.3290 31050779 PMC6503517

[22] Liang KY, Zeger SL Longitudinal data analysis using generalized linear models. Biometrika. 1986;73(1):13-22. doi:10.1093/biomet/73.1.13

[23] Højsgaard S, Halekoh U, Yan J The R package geepack for generalized estimating equations. J Stat Softw. 2006;15(2). doi:10.18637/jss.v015.i02.

[24] Schulz J, DeCamp M, Berkowitz ASA Spending patterns among Medicare ACOs that have reduced costs. J Healthc Manag. 2018;63(6):374-381. doi:10.1097/JHM-D-17-00178 30418364 PMC12706764

[25] Mostashari F, Sanghavi D, McClellan M Health reform and physician-led accountable care: the paradox of primary care physician leadership. JAMA. 2014;311(18):1855-1856. doi:10.1001/jama.2014.4086 24723035

[26] Lewis VA, Colla CH, Carluzzo KL, Kler SE, Fisher ES Accountable care organizations in the United States: market and demographic factors associated with formation. Health Serv Res. 2013;48(6 Pt 1):1840-1858. doi:10.1111/1475-6773.12102 24117222 PMC3876396

[27] Neprash HT, Chernew ME, McWilliams JM Little evidence exists to support the expectation that providers would consolidate to enter new payment models. Health Aff (Millwood). 2017;36(2):346-354. doi:10.1377/hlthaff.2016.0840 28167725 PMC5458639

[28] Yeager VA, Zhang Y, Diana ML Analyzing determinants of hospitals’ accountable care organizations participation: a resource dependency theory perspective. Med Care Res Rev. 2015;72(6):687-706. doi:10.1177/1077558715592295 26156971

[29] Rothman AA, Wagner EH Chronic illness management: what is the role of primary care? Ann Intern Med. 2003;138(3):256-261. doi:10.7326/0003-4819-138-3-200302040-00034 12558376

[30] Barnett ML, Song Z, Landon BE Trends in physician referrals in the United States, 1999-2009. Arch Intern Med. 2012;172(2):163-170. doi:10.1001/archinternmed.2011.722 22271124 PMC3568395

[31] Forrest CB A typology of specialists’ clinical roles. Arch Intern Med. 2009;169(11):1062-1068. doi:10.1001/archinternmed.2009.114 19506176

[32] de Lisle K, Litton T, Brennan A, Muhlestein D The 2017 ACO Survey: what do current trends tell us about the future of accountable care? Health Affairs Blog. https://www.healthaffairs.org/do/10.1377/hblog20171021.165999/full/. Published October 4, 2017. Accessed June 1, 2019.

[33] Mackinney AC, Mueller KJ, McBride TD The march to accountable care organizations-how will rural fare? J Rural Health. 2011;27(1):131-137. doi:10.1111/j.1748-0361.2010.00350.x 21204980

