# Supplementary material for: Association Between Specialist Office Visits and Health Expenditures in Accountable Care Organizations
Source: JAMA Netw Open. 2019 Jul 10;2(7):e196796. doi: 10.1001/jamanetworkopen.2019.6796 (PMC6624801; doi:10.1001/jamanetworkopen.2019.6796)
Supplement: Supplement. — eAppendix. Explanation of Primary Predictor eTable 1. Accountable Care Organization Characteristic Descriptive Statistics, All Groups eTable 2. Per-Beneficiary Person-Year Expenditure Differences Between Specialist Encounter Proportion Groups and Reference (40% to <45%) [file jamanetwopen-2-e196796-s001.pdf]

Supplementary Online Content

Shetty VA, Balzer LB, Geissler KH, Chin DL. Association between specialist office visits and health expenditures in accountable care organizations. *JAMA Netw Open*. 2019;2(7):e196796. doi:10.1001/jamanetworkopen.2019.6796

eAppendix. Explanation of Primary Predictor

eTable 1. Accountable Care Organization Characteristic Descriptive Statistics, All Groups

eTable 2. Per-Beneficiary Person-Year Expenditure Differences Between Specialist Encounter Proportion Groups and Reference (40% to <45%)

This supplementary material has been provided by the authors to give readers additional information about their work.

©2019 Shetty VA et al. *JAMA Network Open*

eAppendix. Explanation of Primary Predictor

The primary predictor used in this study was the proportion of primary care services given by a specialist. This measure was calculated by dividing the total number primary care services given by providers in an ACO (adjusted by person-years) by the number of primary care services given by a specialist. A primary care service is defined as an ambulatory evaluation and management (E&M) service determined by Healthcare Common Procedure Coding System (HCPCS) codes 99201-99215, 99304-99350, G0402, G0438, G0439, and by revenue center codes 0521, 0522, 0524, 0525 when submitted by a federally qualified health center or rural health clinic.[17] If the largest share of a Medicare patient's primary care services are provided by a physician who is a member of an ACO, that patient will be retrospectively attributed to the ACO. Providers aligned with ACOs are identified through tax identification numbers (TINs) and physician type is identified through physician specialty codes. Providers who can give primary care services include primary care physicians (internists, family medicine physicians, geriatricians, and pediatricians), specialists, nurse practitioners, clinical nurse specialists, and physician assistants, and services given at a Federally Qualified Health Center or Rural Health Clinic.

©2019 Shetty VA et al. *JAMA Network Open*

eTable 1. Accountable Care Organization Descriptive Statistics, All Groups

Characteristic	Mean (std. dev)							
	Specialist Encounter Proportion Group[a]							
	**Overall**	**<35%**	**35% to <40%**	**40% to <45%**	**45% to <50%**	**50% to <55%**	**55% to <60%**	**>60%**
**Number of ACO-years**	1836	178	300	431	415	301	147	64
**Number of unique ACOs**	620	59	100	147	136	113	44	21
**Total beneficiary-years**	31861238	1980927	4323048	8120335	8005289	5312818	2911020	1207800
**Expenditures per beneficiary-year ($)**	11017 (2981)	11975 (5611)	10680(2470)	10641 (2566)	10976 (2371)	10932 (2621)	11308 (2533)	12465 (2351)
**ACO Size**	17353 (17046)	11129 (8680)	14410 (11972)	18841 (19935)	19290 (20711)	17651 (15841)	19803 (14758)	18871 (11551)
**Specialist participation[b] (%)**	40.5 (20.5)	29.2 (20.2)	29.2 (20.6)	39.7 (19.5)	39.7 (19.3)	42.1 (20.0)	52.7 (18.4)	63.1 (11.5)
**Proportion of ESRD person-years (%)**	1.0 (0.6)	1.0 (0.9)	1.0 (0.7)	1.0 (0.6)	1.0 (0.5)	1.0 (0.6)	1.0 (0.6)	1.0 (0.6)
**Proportion of Disabled person-years (%)**	14.2 (7.2)	20.3 (9.0)	17.3 (8.0)	14.7 (6.3)	12.9 (5.4)	11.3 (5.1)	10.8 (7.1)	10.3 (4.2)
**Proportion of Dual Eligible person-years (%)**	8.4 (9.7)	13.1 (10.5)	9.9 (10.9)	9.0 (11.8)	6.9 (8.7)	6.4 (6.3)	6.9 (4.8)	6.9 (4.0)
**Proportion of Non-Dual person-years (%)**	76.4 (13.6)	65.6 (15.9)	71.8 (14.5)	75.3 (13.9)	79.2 (11.4)	81.7 (10.1)	81.8 (9.9)	81.8 (7.5)
**ESRD HCC Score[c]**	1.02 (0.06)	1.03 (0.08)	1.03 (0.05)	1.02 (0.05)	1.02 (0.05)	1.01 (0.05)	1.02 (0.05)	1.03 (0.07)
**Disabled HCC Score**	1.09 (0.14)	1.05 (0.22)	1.06 (0.12)	1.09 (0.12)	1.11 (0.11)	1.11 (0.12)	1.11 (0.12)	1.15 (0.20)
**Dual Eligible HCC Score**	1.03 (0.12)	1.01 (0.14)	1.04 (0.11)	1.02 (0.11)	1.04 (0.11)	1.03 (0.12)	1.05 (0.10)	1.06 (0.08)
**Non-Dual HCC Score**	1.06 (0.11)	1.07 (0.21)	1.05 (0.11)	1.05 (0.10)	1.06 (0.10)	1.05 (0.09)	1.06 (0.10)	1.08 (0.08)

Abbreviations: ACO, accountable care organization; ESRD, end-stage renal disease; HCC, hierarchical condition category

[a] Specialist encounter proportion is the proportion of office visits provided by a specialist

[b] Specialist participation is the proportion of clinicians participating in the ACO who were specialists

[c] Higher HCC scores indicate lower health status; the HCC risk scores for each enrollment type are renormalized to their own populations and thus are not on the same scale and not comparable across eligibility types

eTable 2. Per-Beneficiary Person-Year Expenditure Differences Between Specialist Encounter Proportion Groups and Reference (40% to <45%)

	**Specialist Encounter Proportion Groups**						
	**<35%**	**35% to <40%**	**40% to <45%**	**45% to <50%**	**50% to <55%**	**55% to <60%**	**≥60%**
**Unadjusted estimate ($)**	1200.8*	-15.7	-	82.4	152.2	350.1	720.3
**95% CI**	77.4, 2324.0	-382.5, 351.0	-	-244.2, 409.0	-330.6, 635.0	-260.4, 961.0	-119.7, 1560
**Adjusted estimate ($)**	1129.0**	76.9	-	13.1	256.1	229.1	752.1*
**95% CI**	444.5, 1814.0	-215.0, 368.7	-	-262.7, 265.3	-90.6, 602.7	-239.5, 697.7	115.2, 1389.0

Abbreviations: CI, confidence interval

Specialist encounter proportion is the proportion of office visits provided by a specialist

*Significant at $P < 0.05$; **Significant at $P < 0.01$; ***Significant at $P < 0.001$

©2019 Shetty VA et al. *JAMA Network Open*
